# High frequency input impedance modeling of low-voltage residential installations - influence on lightning overvoltage simulations results

**DOI:** 10.1186/2193-1801-3-690

**Published:** 2014-11-25

**Authors:** Welson Bassi

**Affiliations:** Institute of Energy and Environment (IEE), University of São Paulo (USP), Av. Prof. Luciano Gualberto 1289 CEP, 05508-900 São Paulo, SP Brazil

**Keywords:** Lightning, Power distribution, Power distribution lines, Power system transients, Low-voltage, Surge protection

## Abstract

The overvoltage level of a system is strongly dependent on the connected loads and with more precise models, better and more reliable simulation results are obtained. This paper presents the input impedance characteristics, measured over a wide range of frequencies, of various actual low-voltage residential installations. The measured frequency responses were fitted by effective RLC models and a general model was also developed. The range of frequencies considered in the study, nearly d.c. up to 5 MHz, allows the use of these models for lightning or switching studies. It is also presented overvoltage simulations, using different residential installations models presented in the paper, of a distribution network subjected to lightning surges on the medium voltage circuit.

## Background

Surges caused by lightning or switching events can cause upset or damage on electrical and electronic equipment inside low-voltage installations. The increasing utilization of sensitive equipment enhanced the problem over the last recent years and, as a consequence, more relevance is being given to the actions involving protection of such equipment.

Digital simulation using transient computational software is a powerful tool for evaluation of the transient levels of a certain system or network and alternatives for overvoltage mitigation. However, the simulations demand for using models to adequately represent all the components of the system under observation.

The actual behavior of the overvoltages in a system or installation is strongly dependent on the connected loads and with more precise models, more realistic and reliable simulation results are obtained.

Overvoltage studies can present a considerable variety of possibilities of modeling of low-voltage power installations (LVPI) in distribution systems, either in laboratory experimental setups or in digital simulations. In (Mcmillen et al. 1988; Dugan and Smith 1988; Smith and Puri 1988; Goedde et al. 1992; Hosfet et al. 1992; Standler 1992 and Mirra et al. 1997) the consumers are represented by lumped resistances or capacitances or simple association of them. In (Borghetti et al. 2005) it was used the matching impedance of the low-voltage line for representing the consumers. In (Sekioka et al. 2010a, Sekioka et al. 2010b) the home installations were represented by the surge protective devices (SPD) in them. A model based on real measurements of a sole installation was used when simulating the induced voltages on low-voltage networks in (Hoidalen 1998).

The representation of the consumer installations connected to the secondary distribution networks really presents difficulties that make virtually impossible to draw up absolute models perfectly fitted to the conditions found in reality. However, it is possible to obtain simple models adjusted to an overall behavior observed in tests and measurements.

This paper aims to present the models development and a sensitive evaluation of overvoltage at LVPI entrances, in the case of a direct lightning strike to the medium voltage (MV) network, depending upon the adopted model for the low-voltage consumer installations.

## Methods

**Figure 1:**
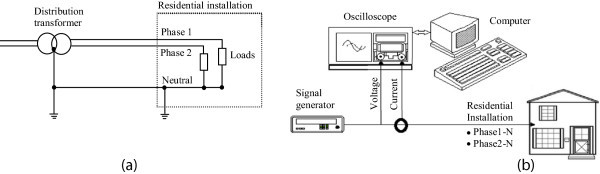
**General conditions. (a)** Ground system (TN) and wiring on the evaluated installations **(b)** Input impedance measurement test setup.

Residential installations were selected and their input impedances were measured over a range of frequencies from nearly d.c. up to 5 MHz considering both magnitude and phase. The ground configuration in all tested buildings is the TN system, as shown in Figure 1a. The measurements were performed at the entrance electric board, using common equipment to build up an impedance analyzer, mainly consisting of a signal generator (20 MHz, maximum output voltage 5 V rms) and a digital oscilloscope (8 bits, 100 MHz) as can be seen in Figure 1b. The current drained by the installation under test was measured by a current probe/amplifier set (d.c. to 50 MHz). The signals were acquired by a computer with software developed to compute the magnitude and phase values of the impedance.

The installation under test was disconnected from the a.c. utility power system remaining all the fixed appliances (e.g. TV, refrigerator, stereo, desktop computer etc.) connected to the internal outlets. This powered off condition of the house equipment represents just some slight variations on their input impedance for frequencies up to 10 MHz (Chen et al. 2011).

Five low-voltage installations, with essentially the same constructive arrangement, 2.5 mm^2^ to 4 mm^2^ PVC insulated conductors through conduits inside brick walls, were tested:

Installation #1, apartment with approximately 90 m^2^, with the following equipment connected: refrigerator, microwave oven, dish washer machine, washer machine, wireless telephone, TV set 29”, TV set 14”, desktop computer, stereo and DVD player;

Installation #2, house with approximately 120 m^2^, with the following equipment connected: refrigerator, microwave oven, washer machine, TV set 20”, stereo and DVD player;

Installation #3, apartment with approximately 60 m^2^, with the following equipment connected: refrigerator, microwave oven and TV set 20”;

Installation #4, house with approximately 150 m^2^, with the following equipment connected: refrigerator, freezer, microwave oven, dish washer machine, washer, wireless telephone, TV set 29”, TV set 20”, desktop computer, stereo and DVD player;

Installation #5, apartment with approximately 75 m^2^, with the following equipment connected: refrigerator, microwave oven, TV set 20” and washer.

## Results

### Input impedance measurement and models

**Figure 2:**
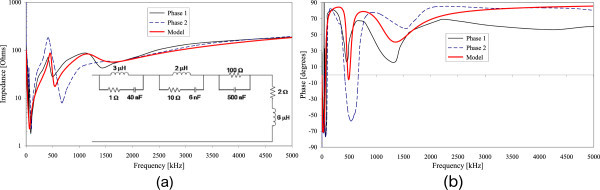
**Input impedance of Installation #1 (a) Magnitude (b) Phase.**

**Figure 3:**
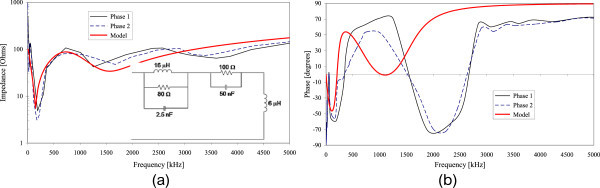
**Input impedance of Installation #2 (a) Magnitude (b) Phase.**

**Figure 4:**
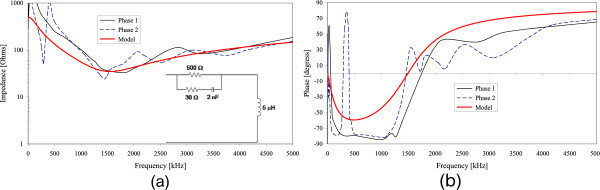
**Input impedance of Installation #3 (a) Magnitude (b) Phase.**

**Figure 5:**
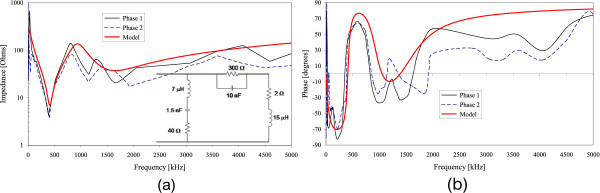
**Input impedance of Installation #4 (a) Magnitude (b) Phase.**

**Figure 6:**
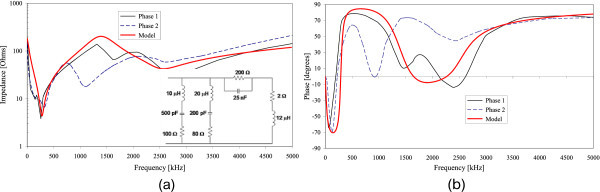
**Input impedance of Installation #5 (a) Magnitude (b) Phase.**

Figures 2, 3, 4, 5 and 6 show the results of the measured input impedance (magnitude and phase) for each installation and the calculated curves using the respective models.

The models were developed observing the break frequencies on the impedance frequency curves and adjusting them accordingly using RLC elements or groups for reproducing those frequencies.

Due to the variety of internal configurations there are dissimilarities on the measured input impedance among the several installations. However, a similar overall behavior of the curves is observed: in the range of lower frequencies the input impedance has predominantly a capacitive characteristic and for higher frequencies the impedance presents an inductive behavior.

This aspect of the input impedance over the range of frequencies up to 5 MHz can be taken into consideration for proposing an approximate and simple general model for the installations, to be used in the computational simulations.

Because of the large spread among the curves, a certain approach is necessary for averaging them before fitting a model for the set. One could suggest several numerical methods, but a simple and feasible approach is the calculation of the harmonic mean of all impedance magnitude values for every frequency component. The harmonic mean is best used in situations where extreme outliers exist in the population giving less significance to high and low outliers values and providing a proper representation of the average. The harmonic mean *H* of *n* positive real numbers *x*_*1*_*, x*_*2*_*, …, x*_*n*_ is defined:
1

**Figure 7:**
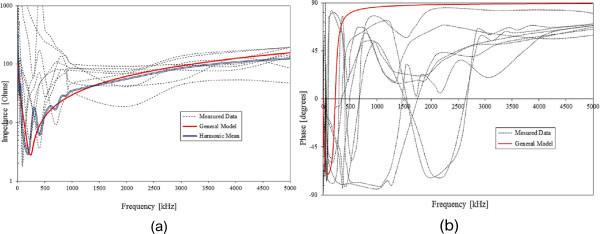
**Input impedance curves. (a)** Measured and calculated magnitude (using the harmonic mean and the model of Figure 8) **(b)** Measured and calculated phase (using the model of Figure 8).

**Figure 8:**
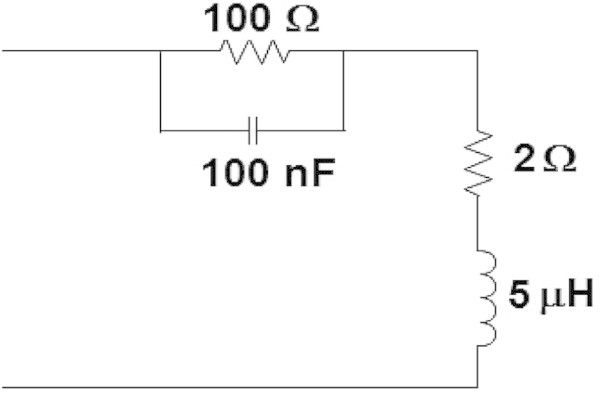
**General model.**

Figure 7 shows all the measured impedance curves together, the harmonic mean curve of the magnitude and the calculated curves using the general RLC model shown in the Figure 8, which was developed observing the behavior of the averaged impedance curve.

### Application

In order to evaluate the influence of the consumer’s representation, computational simulations were performed with different models for representing the consumer’s installations of a low-voltage distribution system. The overvoltages caused by direct impact of lightning on the medium voltage circuit were calculated, using the ATP – Alternative Transient Program.

**Figure 9:**
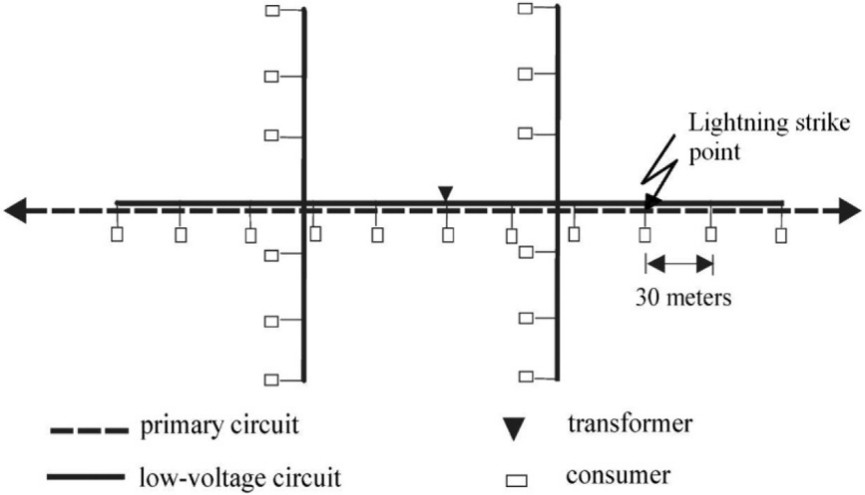
**Distribution system topology used in the simulations.**

**Figure 10:**
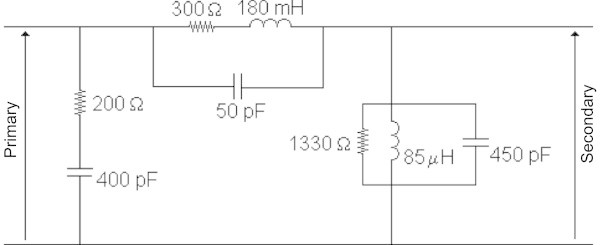
**Distribution transformer model (per phase) used in the simulations.**

The simulation of distribution systems is particularly difficult due to the variety of configurations and components, but a typical and complete system was modeled taking also into consideration the occurrence of flashovers at the medium voltage insulators. The topology overview is shown in Figure 9 and it is considered to be representative: a straight (13.8 kV) primary circuit and a low-voltage network (127V/220V) with a portion coupled to the primary and two uncoupled laterals. It was also considered the characteristics of the conductors as well as the models used for representing the distribution transformer and the surge arresters. The distribution transformer model used for representing high frequencies, shown in the Figure 10, was developed using frequency response analysis, taking into account the load conditions, as described in detail in [Piantini et al. 1999. The lightning stroke current is injected into the primary line, at the point shown in Figure 9, and was represented by a triangular waveshape with amplitude of 45 kA, time to peak of 2 μs and time to half value of 80 μs. Resistances of 100 Ω, connected to reference earth, were considered for grounding poles and consumer’s installations. So, the representation of grounding using lumped resistances is not the most possible accurate model, but due to the complexity of the overall simulated system, this simplification was adopted, and the final results tend to be more conservative. The complete description and details is in (Bassi and Janiszewski 2003).

Different models for the consumer installations were used as shown in Table 1 in order to evaluate the overvoltage system responses. The cases varied among the developed models, simple lumped resistances of 100 Ω, inductances of 5 μH, capacitances of 4 nF as used in (Mirra et al. 1997) and the TN model presented in (Hoidalen 1998).

**Table 1:** **Simulated cases**

Simulation case	Consumers installation model
#1	installation #1 as per Figure 3
#2	installation #2 as per Figure 4
#3	installation #3 as per Figure 5
#4	installation #4 as per Figure 6
#5	installation #5 as per Figure 7
#6	general model as per Figure 9
#7	TN model
#8	resistance of 100 Ω
#9	capacitance of 4 nF
#10	inductance of 5 μH

Figures 11, 12, 13, 14, 15, 16, 17, 18, 19 and 20 show simulation results for the phase to neutral overvoltage values at consumer’s entrances expressed in different colored voltage ranges, considering the phase with the higher overvoltage value. The observed waveforms at consumer installation are also presented at some points of the low-voltage system.

**Figure 11:**
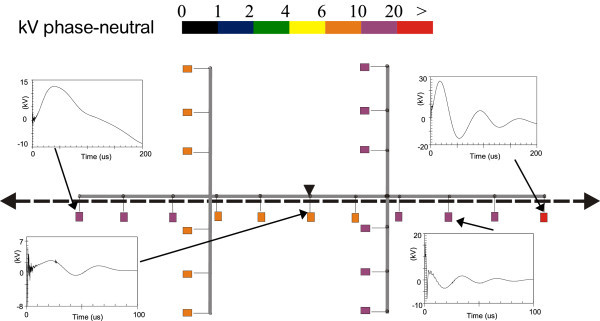
**Simulation results for case #1.**

**Figure 12:**
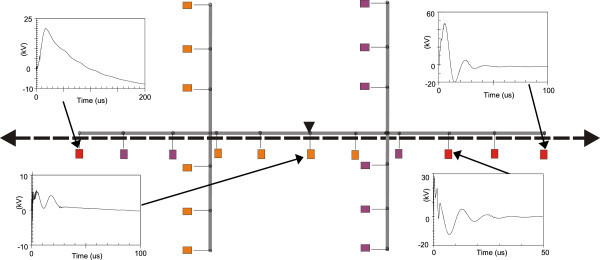
**Simulation results for case #2.**

**Figure 13:**
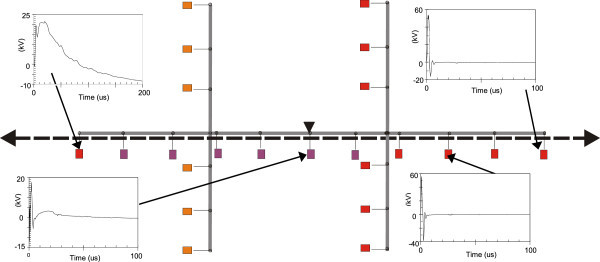
**Simulation results for case #3.**

**Figure 14:**
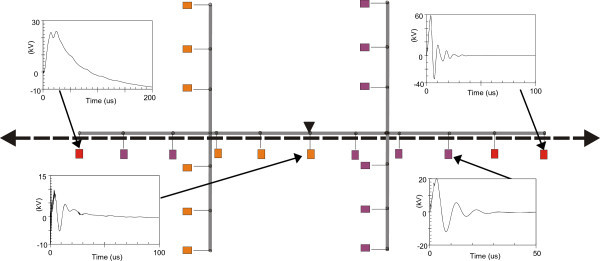
**Simulation results for case #4.**

**Figure 15:**
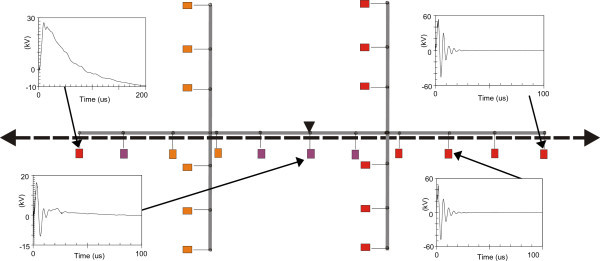
**Simulation results for case #5.**

**Figure 16:**
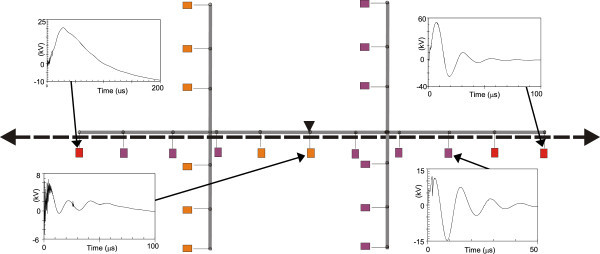
**Simulation results for case #6.**

**Figure 17:**
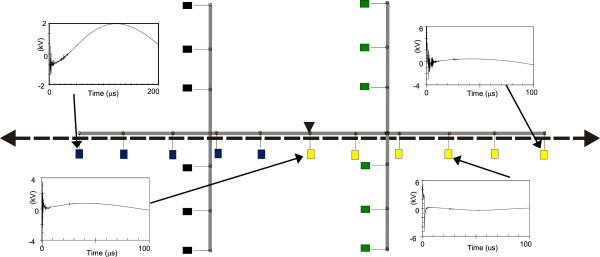
**Simulation results for case #7.**

**Figure 18:**
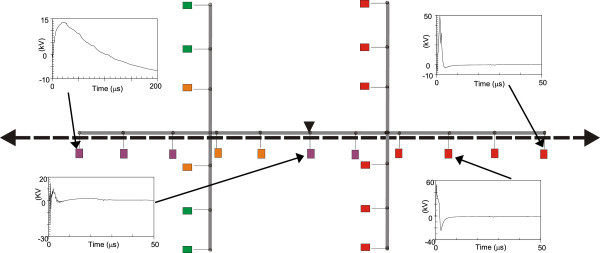
**Simulation results for case #8.**

**Figure 19:**
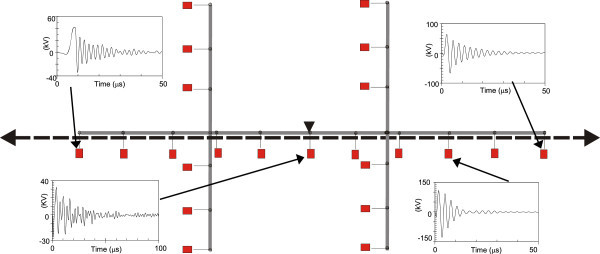
**Simulation results for case #9.**

**Figure 20:**
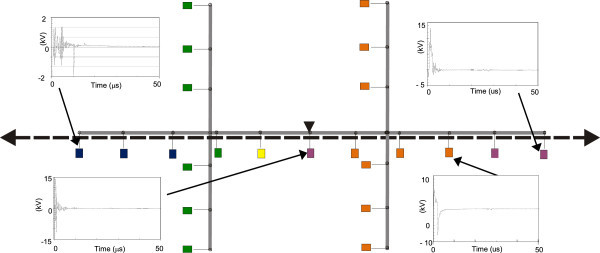
**Simulation results for case #10.**

The frequency of occurrence of the overvoltage values in all consumers’ entrances (in both phases) is summarized in Table 2, which shows the values exceeded in 90%, 50% and 10% of the consumer points in each simulation case. It can be observed, by visual inspection of Figures 11, 12, 13, 14, 15, 16, 17, 18, 19 and 20 and Table 2, the great dependency of the resulting overall overvoltage scenario upon the adopted model for the low-voltage installations.

**Table 2:** **Frequency distribution of the number of overvoltage observations where the indicated values are exceeded**

Simulation case	90%	50%	10%
#1	4 kV	6 kV	10 kV
#2	2 kV	6 kV	10 kV
#3	4 kV	6 kV	10 kV
#4	4 kV	6 kV	10 kV
#5	4 kV	10 kV	20 kV
#6	4 kV	6 kV	10 kV
#7	1 kV	1 kV	6 kV
#8	2 kV	10 kV	20 kV
#9	6 kV	20 kV	20 kV
#10	1 kV	2 kV	6 kV

Despite the differences in the waveforms, the frequencies of occurrence of the overvoltage peak values for cases #1 to #6 are similar. Thus, the general model used in case #6 can be considered as representative of the particular group of measured installations of cases #1 to #5.

One can observe the oscillatory behavior in the calculated overvoltages of cases #1 to #6, with the dominant characteristics regarding the first rise time and range of frequency quite similar to the damped oscillatory ring wave test waveform defined in (IEEE The Institute of Electrical and Electronics Engineers 2002) which was prescribed after evaluating extensive data collection of real surge recordings during several years in many installations. This similarity leads to presume a realistic scenario for the calculated results using models based on measurements.

For the resistive model of case #8 (Figure 18), the calculated overvoltages do not present significant oscillations, given the dissipative character of the loads. The model of case #7 (Figure 17) and the lumped inductance of 5 μH of case #10 (Figure 20) leads to smaller peak values, and, on the contrary, for the model of case #9 (Figure 19) the overvoltages present high values and oscillations of elevated frequency, with a significantly different behavior from that of the other models.

**Figure 21:**
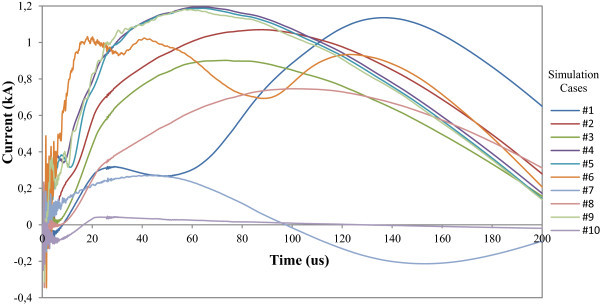
**Simulation results of maximum currents at transformer secondary.**

The calculated drained surge currents in the secondary circuit are sensitively smaller than the injected lightning current (Bassi and Janiszewski 2003). As illustration, Figure 21, shows the maximum currents through the conductors of the transformer secondary for all the simulated cases (#1 to #10). It can be seen the great diversity of resulting waveshapes, but with peaks values in the range up to about1 kA.

If parallel buildings are supplied by the same supply transformer this will decrease the overall earthing impedance of the low voltage supply system. A lower earthing impedance of the low voltage supply system could results in higher impulse currents flowing on it.

## Conclusions

Models for representing residential low-voltage installations were developed. These models can be used in transient simulations software to evaluate switching and lightning overvoltages in distribution systems.

A particular or individual installation can be modelled with good matching with the measurements. Modelling all individual installation will not be feasible, but general simple RLC models for groups or installations categories would be reasonable to be developed, as the presented general proposed model.

It must be emphasized that modelling of consumer’s installations is not a simple task and involves difficulties: the large variety of configurations and equipment and the dynamic behavior of the loads intraday and due to seasonality.

The results presented in this study, in some cases, show a remarkable variation of peak values and the resulting waveforms for different models affecting the overvoltage evaluation of the system under observation. This aspect justifies the importance of utilization of models based on real measurements which tends to lead more realistic results and increases the reliability of the simulation processes.
